# Supplementary material for: Natural quantum reservoir computing for temporal information processing
Source: Sci Rep. 2022 Jan 25;12:1353. doi: 10.1038/s41598-022-05061-w (PMC8789868; doi:10.1038/s41598-022-05061-w)
Supplement: Supplementary file 1 — Supplementary Table 1. [file 41598_2022_5061_MOESM1_ESM.pdf]

Natural quantum reservoir computing for temporal information processing

Yudai Suzuki[1,*], Qi Gao[2,3], Ken C. Pradel[2], Kenji Yasuoka[1], and Naoki Yamamoto[3,4]

[1]Department of Mechanical Engineering, Keio University, Hiyoshi 3-14-1, Kohoku, Yokohama 223-8522, Japan
[2]Mitsubishi Chemical Corporation, Science & Innovation Center, 1000, Kamoshida-cho, Aoba-ku, Yokohama 227-8502, Japan
[3]Quantum Computing Center, Keio University, Hiyoshi 3-14-1, Kohoku, Yokohama 223-8522, Japan
[4]Department of Applied Physics and Physico-Informatics, Keio University, Hiyoshi 3-14-1, Kohoku, Yokohama 223-8522, Japan
[*]email: yudai.suzuki.sh@gmail.com

Supplementary Table

Table 1. The global average of NMSEs and the global minimum of NMSEs of the echo state networks (ESNs) for the NARMA tasks. The number of internal nodes of ESN changes as $N_{ESN} = 2, 5, 10, 20, 50$ for each NARMA task. As for the global minimum of NMSE, the optimal spectral radius of W is also shown.

Task	Model	the global average of NMSE		the global minimum of NMSE		
		Mean	Std	Mean	Std	Spectral radius of W
NARMA2	ESN ($N_{ESN} = 2$)	1.3×10^{-5}	1.3×10^{-5}	8.9×10^{-6}	1.1×10^{-5}	0.01
	ESN ($N_{ESN} = 5$)	3.5×10^{-6}	7.6×10^{-6}	1.4×10^{-6}	4.5×10^{-6}	0.01
	ESN ($N_{ESN} = 10$)	7.6×10^{-7}	2.0×10^{-6}	1.5×10^{-7}	7.6×10^{-8}	0.01
	ESN ($N_{ESN} = 20$)	1.7×10^{-7}	6.0×10^{-7}	2.4×10^{-8}	1.8×10^{-8}	0.14
	ESN ($N_{ESN} = 50$)	1.9×10^{-7}	1.0×10^{-6}	2.1×10^{-9}	1.9×10^{-9}	0.47
NARMA5	ESN ($N_{ESN} = 2$)	1.8×10^{-3}	8.5×10^{-4}	1.5×10^{-3}	2.5×10^{-4}	0.01
	ESN ($N_{ESN} = 5$)	4.9×10^{-4}	1.1×10^{-3}	2.1×10^{-4}	4.3×10^{-4}	0.23
	ESN ($N_{ESN} = 10$)	1.1×10^{-4}	2.9×10^{-4}	2.7×10^{-5}	1.7×10^{-5}	0.18
	ESN ($N_{ESN} = 20$)	1.9×10^{-5}	9.7×10^{-5}	4.3×10^{-6}	1.4×10^{-6}	0.13
	ESN ($N_{ESN} = 50$)	1.2×10^{-5}	2.5×10^{-5}	2.4×10^{-6}	2.3×10^{-6}	0.05
NARMA10	ESN ($N_{ESN} = 2$)	1.3×10^{-3}	7.2×10^{-4}	1.2×10^{-3}	6.4×10^{-4}	0.01
	ESN ($N_{ESN} = 5$)	7.7×10^{-4}	5.9×10^{-4}	5.7×10^{-4}	3.4×10^{-4}	0.23
	ESN ($N_{ESN} = 10$)	4.2×10^{-4}	4.1×10^{-4}	2.6×10^{-4}	2.2×10^{-4}	0.50
	ESN ($N_{ESN} = 20$)	2.6×10^{-4}	2.5×10^{-4}	1.8×10^{-4}	9.0×10^{-5}	0.64
	ESN ($N_{ESN} = 50$)	1.0×10^{-4}	2.0×10^{-4}	4.9×10^{-5}	3.8×10^{-5}	0.68
